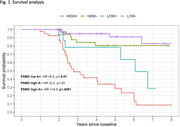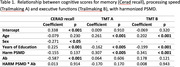# The role of Peak width Skeletonized Mean Diffusivity in AD disease progression

**DOI:** 10.1002/alz.095692

**Published:** 2025-01-09

**Authors:** Jonas Alexander Jarholm, Sandra Tecelao, Lene Pålhaugen, Bjørn‐Eivind Kirsebom, Atle Bjornerud, Tormod Fladby, Per Selnes

**Affiliations:** ^1^ Institute of Clinical Medicine, University of Oslo, Oslo, Oslo Norway; ^2^ Akershus University Hospital, Nordbyhagen, Akershus Norway; ^3^ Faculty of Health Sciences, UiT The Arctic University of Norway, Tromsø, Troms Norway; ^4^ University Hospital of Northern Norway, Tromsø, Troms Norway; ^5^ Department of Physics, University of Oslo, Oslo, Oslo Norway; ^6^ Department of Diagnostic Physics, Oslo University Hospital, Rikshospitalet, Oslo, Oslo Norway

## Abstract

**Background:**

Cerebral small vessel disease (CSVD) is common in Alzheimer’s disease (AD), but it is unclear how CSVD affects cognition and disease progression. Peak width of Skeletonized Mean Diffusivity (PSMD) is a Magnetic Resonance Imaging (MRI) marker of global white matter integrity, believed to reflect both total vascular burden and the cognitive impact of CSVD. We examined the relationship between PSMD and memory, processing speed and executive function, and to assess the predictive value of PSMD on clinical progression.

**Method:**

265 cases and controls between 40‐80 years old were recruited from the Norwegian multi‐center study DDI (Dementia Disease Initiation), with 2‐5 follow ups (0.7‐8 years). Subjects were according to the Clinical Dementia Rating (CDR), with 0 as cognitively unimpaired (CU) if, 0,5 as mild cognitive impairment (MCI) if CDR, and ≥ 1 as dementia. Amyloid status was determined from Amyloid‐PET or from amyloid‐β42/40. PSMD was obtained from 6 MRI scanners and harmonized for scanner effects in R using ComBat, with age, sex, staging and amyloid status at baseline as covariates.

The relationship between PSMD and cognitive scores was assessed using a general linear model, with age, sex, education and an PSMD‐amyloid interaction variable as covariates. Subjects were classified into the following groups: A‐ low PSMD, A+ low PSMD, A‐ low PSMD, A+ high PSMD high, where PSMD high or low signifies above or below the group median. Survival analysis was performed in 195 subjects, using the “survival” package from R, with progression from CU to MCI/dementia or from MCI to dementia as the events of interest. Hazard ratios were assessed with the Cox proportional‐hazards model with age and A status/PSMD at baseline and sex as covariates.

**Result:**

There was a significant relationship (p<0.01) between PSMD and TMT A and B, but not with CERAD recall. PSMD low A+ subjects had 5.5 times higher risk of cognitive decline (p<0.01) than PSMD low A‐, while PSMD high A+ had 14.3 times higher risk.

**Conclusion:**

We found that high PSMD load increases the risk of AD disease progression, but is not significantly associated with delayed recall, the core cognitive biomarker of AD.